# Teaching Medical Students in a New Rural Longitudinal Clerkship: Opportunities and Constraints

**DOI:** 10.29024/aogh.17

**Published:** 2018-04-30

**Authors:** Marietjie de Villiers, Hoffie Conradie, Susan van Schalkwyk

**Affiliations:** 1 Faculty of Medicine and Health Sciences, Stellenbosch University, ZA

## Abstract

**Background:**

Medical schools in Africa are responding to the call to increase numbers of medical graduates by up-scaling decentralized clinical training. One approach to decentralized clinical training is the longitudinal integrated clerkship (LIC), where students benefit from continuity of setting and supervision. The ability of family physician supervisors to take responsibility for the clinical training of medical students over a longer period than the usual, in addition to managing their extensive role on the district health platform, is central to the success of such training.

**Objective:**

This study investigated the teaching experiences of family physicians as clinical supervisors in a newly introduced LIC model in a rural sub-district in the Western Cape, South Africa.

**Method:**

Nine semi-structured interviews were conducted with six family physicians as part of the Stellenbosch University Rural Medical Education Partnership Initiative (SURMEPI) five-year longitudinal study. Code lists were developed inductively using Atlas.ti v7, they were compared, integrated, and categories were identified. Emerging common themes were developed.

**Findings:**

Three overarching themes emerged from the data, each containing subthemes. The rural platform was seen to be an enabling learning space for the LIC students. The family physicians’ experienced their new teaching role in the LIC as empowering, but also challenging. Lack of time for teaching and the unstructured nature of the work emerged as constraints. Despite being uncertain about the new LIC model, the family physicians felt that it was easier to manage than anticipated.

**Conclusion:**

The centrality of the rural context framed the teaching experiences of the family physicians in the new LIC, forming the pivot around which constraints and opportunities for teaching arose. The African family physician is well positioned to make an important contribution to the upscaling of decentralized medical training, but would need to be supported by academic institutions and health service managers in their teaching role.

## Background

Medical schools in Africa are responding to the call for increasing the numbers of medical graduates by upscaling decentralized clinical training [1,2,3]. Many of these initiatives have been prompted by the President’s Emergency Plan for AIDS Relief (PEPFAR)-funded Medical Education Partnership Initiative (MEPI) [4]. Although evidence from these efforts is emerging [1,5], there is a need to understand how the context at these distant sites influences clinical training outcomes. Part of this context relates to the nature of the supervision provided, and how it can be optimized. This is crucial to the sustained success of clinical training that occurs away from the tertiary training complex [6].

One approach to decentralized clinical training is the longitudinal integrated clerkship (LIC), where medical students benefit from continuity of the clinical setting and supervision, most typically for a year at a time [7]. The LIC model is seen as an innovative way to do clinical training, while also supporting workforce challenges – such as recruitment and retention of medical doctors for rural and primary care practice [8,9,10]. Stellenbosch University introduced the first LIC model in southern Africa in 2011 as part of the establishment of the Ukwanda Rural Clinical School (RCS) [11]. Students can choose to work at a rural district health complex for the entire final year of their studies. This model is classified as a comprehensive LIC [7], where students learn by participating in the context of undifferentiated and continuous care at the district hospital and at primary health care clinics (PHCs) in the district. A specialist family physician is primarily responsible for their supervision and learning for the year, supported by regular visits by medical specialists from the regional hospital [11,12].

In South Africa, rural health districts are divided into a number of sub-districts each responsible for a defined population. In rural Western Cape, a health sub-district with a population of approximately 100,000 people will typically comprise a rural district hospital with 40 to 80 beds and five to eight community health centers (CHC) or PHC clinics. The family physician based at the district hospital is responsible for healthcare in the sub-district and is supported by five to ten generalist doctors (“medical officers”), depending on the size of the district. At the CHC and PHC clinics, first-contact care is provided by clinical nurse practitioners. The district hospital provides acute care, adult and pediatric in-patient care, obstetric care and theatre, X-ray, ultrasound, and laboratory services. The district hospital refers patients to a regional hospital with resident specialists.

Family physicians have been employed in the district health system in the Western Cape since 2011. District managers suggest that these family physicians impact significantly on the quality of clinical care and the health system’s performance [13]. Emerging evidence suggests that family physicians are essential in strengthening clinical care and governance at district hospitals, reducing referrals to higher levels of care, and enhancing community-oriented care [14,15]. Capacity-building has been identified as a critical role for the African family physician [16]. The ability of young, newly qualified family physicians to take responsibility for the clinical training of medical students over a period of a year, in addition to fulfilling a substantial clinical role in a district hospital, is key to the success of such LIC training models [10,17].

## Objective

This study conducted a qualitative exploration into the teaching experiences of clinical teachers on a new rural teaching platform, specifically aiming to investigate the experiences of family physicians as clinical supervisors in a newly introduced LIC model in a rural sub-district in the Western Cape, South Africa. The study explored the following research questions: How did the family physicians experience their unique teaching responsibilities in a new LIC model? What were the opportunities and challenges that they encountered?

## Method

The study method is reported here, following the consolidated criteria for reporting qualitative research (COREQ) guidelines, namely research team and reflexivity, study design, and analysis and findings [18]. The first and second authors are both family physicians, whilst the third author is an educationalist who conceptualized and led the overarching study. The second author worked with the participants in the RCS, whilst the first and third authors were not directly involved with the participants.

This study was situated in a five-year longitudinal study on the implementation of the RCS [11]. The over-arching study was set in in an interpretive paradigm. Purposive sampling was done to include all six family physicians who were working at the RCS at the time. Nine semi-structured interviews were conducted following a longitudinal approach over time with interviews conducted during the first year of the RCS, and again two to three years later (Table 1).

**Table 1:** Longitudinal Semi-Structured Interviews with Family Physicians.

	2011	2012	2014	2015

**FP – 01**	√		√	
**FP – 02**		√	√	
**FP – 03**		√	√	
**FP – 04**		√		
**FP – 05**			√	
**FP – 06**				√

The average age of the family physicians at the start of the study was 35 (range 34–37). They had been qualified as family physicians for between zero and three years when they first started supervising the students on the platform. Two of the six family physicians were female.

The interviews were held either telephonically or face-to-face, depending on the availability of the interviewee, in the interviewee’s language of choice (English or Afrikaans). The interviews lasted about 30 minutes and were audiotaped and transcribed. A professional language translator later translated Afrikaans quotations to English. The translations were quality checked by the authors who are all proficient in both languages.

Thematic analysis was done following the six steps described by Braun and Clarke [19]. These steps are data familiarizing, coding, searching for themes, reviewing themes, defining and naming themes, and reporting the data. The authors read the transcripts several times to familiarize themselves with the data. Coding was done inductively using Atlas.ti v7 (http://www.atlasti.com) to allow key points to emerge from the data. The authors’ code lists were compared and common categories identified where after the code lists were integrated. Through discussions with all three authors, common themes emerging from the final code list were identified and described. The datasets were subsequently revisited to extract appropriate quotes, while validating the themes at the same time. All the authors scrutinized this final process in order to enhance the rigor of the analysis. Ethics approval was obtained from the Research Ethics Committee at Stellenbosch University Faculty of Medicine and Health Sciences, and the ethics approval number N11/07/245 was awarded.

## Findings

Three themes were developed from the data, namely: the district health platform as a place for student learning; teaching on the district health platform; and enablers and challenges of training on the district health platform. See Table 2 for a summary of the themes and the subthemes. Quotations illustrative of each of the themes are provided in this section and are numbered according to the responding family physician and the year of the interview. Afrikaans quotations were translated into English by a professional translator and indicated by a (T) after the quotation.

**Table 2:** Results – Themes and Subthemes.

Themes	Subthemes

The district health platform as a place for student learning	Patient profile and approach to patient care
	Contribution to health service and community
	Student attributes that facilitate learning in this context
Teaching on the district health platform	Responsibility as teacher
	Enriching and stimulating experience
	Participation of other staff
Enablers and challenges for training on the district health platform	Opportunity to teach
	Structure and organization
	Uncertainty
	Time

### The District Health Platform as a Place for Student Learning

The district health platform was seen to be an enabling learning space, particularly with regard to patient interaction and community engagement. In this context, the students’ own disposition towards learning was seen to be of relevance.

**Patient profile and approach to patient care:** The interviewees described learning opportunities on the district platform with students encountering patients with common conditions presenting in an undifferentiated manner.

FP03-2014 Because anything can walk into our casualty, it’s not only surgery or pediatrics. So they (the students) have to see the patient as a whole and decide what it is. They get first-hand experience with anything really, from tonsillitis, and a lot of things they don’t see at X (tertiary hospital). (T)

FP06-2015 It’s a busy hospital, it’s a busy pathology, it’s a busy maternity ward, it’s a bit of everything. So here they are, I think, [given] good exposure to health and all the types of pathologies that have to prepare them for their exams and their future. (T)

FP01-2011 I think they learn to work very effectively to handle everything. Whatever comes through the door, they’ll learn to handle it, and stabilize it and they’ll be able to refer very appropriately. (T)

The students had the opportunity to manage patients from admission to discharge – and beyond into the community. This meant that they were exposed to the patients’ home environment within the community.

FP02-2014 They (the students) are part of the community … also the fact that they lived [here] and they interacted with the people in the hospital, but also outside the hospital. (T)

FP02-2012 That it’s not only the approach to a headache, but the approach to [the] headache of the person here in front of me … that they (the students) see more of the patient in his context. (T)

**Contribution to health service and community:** The interviewees described the positive contribution that the students made to patient care and service provision, with patients expressing their gratitude towards the students.

FP06-2015 They help, they see the patient in the meantime, they do an examination and stuff for me and then they come and present the patient to me. Especially if it’s so chaotic. It makes it easier, because then they’ve already done it. (T)

FP05-2014 The patients actually feel they get better service, because the students spend more time with them. (T)

The family physicians emphasized students’ involvement and acceptance in the community, and described feeling proud of the students.

FP06-2015 They (the students) went to talk there (at the high school) with the children and her with the girls and him with the boys, and the feedback afterwards was very high. The number of children who contacted her, especially, talked about sexual health and prevention [of] pregnancies and now also treatment, STIs and whatnot, and HIV and everything … even asked [told] the parents to come and talk to them. (T)

**Student attributes that facilitate learning in this context:** The interviewees suggested that students who are responsible for their own learning and prepared to work hard and embrace professionalism were a good fit for training on the platform.

FP01-2015 So they actually are a little different to your normal student, because they have to take so much responsibility and learn on their own and take responsibility for what they do here. So I think they function differently from the standard student. (T)

The family physicians emphasized that students should look for learning opportunities and embrace professionalism, and – in doing so – develop confidence.

FP03-2014 They’re very professional with the patients and ethical and good. The more self-confidence they have, of course, they also convey that to the patients. They’ll tell the patient more with confidence what he must do and mustn’t do, and they know exactly what to say. But they had very good bedside manners and things like that from the beginning. (T)

### Teaching on the district health platform

The family physicians shared their thoughts on being a ‘teacher’ on the district health platform, describing the experience as enabling and enriching. This new teaching role brought with it challenging responsibilities, but also offered opportunities.

**Responsibility as teacher:** The family physicians were performing clinical, outreach, and training duties, and thus had a full workload. Nevertheless, they felt responsible for the students’ training and assessment outcomes, and were aware of how these outcomes might reflect on their own practice.

FP01-2011 It was a sort of difficult year for me in the sense that I felt very responsible for the students. It’s sort of if they struggle, then it reflects on me almost. (T)

**Enriching and stimulating experience:** In spite of being aware of the responsibility, however, the family physicians enjoyed being involved with students over an extended period. They acquainted themselves with the students as individuals, accepting them as part of the team. They were proud to be involved in the new LIC implementation.

FP05-2014 It’s often there where I have very rich interaction with the students, and we get fantastic patients who create tremendous learning opportunities. We had a patient the other day with a polyarthritis, an asymmetrical polyarthritis, and it drew tremendously rich discussion. (T)

FP02-2014 There were always times when you actually are with students that you realize what you missed the other times, because it’s tremendously stimulating to work with them. I think what’s also nice for me about the longitudinal model is that continuity. I realize you have a relationship with the people, you know the student as an individual, as a person, and you know what his strong points are and you know how to, what to take with a touch of humor and what to address on a more serious level. (T)

The family physicians found the students to be enthusiastic, inspiring, and stimulating. They felt the students kept them “on their toes,” which led them to update their own knowledge and skills.

FP03-2014 It’s nice to have such new, young students. They’re very positive and that’s nice. I like that. It’s not just like us that we see every day. They’re new faces, so I think they influence the whole, personally, everyone in a positive way. They also teach us new things, and we have to stay on our toes. (T)

**Participation of other staff:** Other staff in the hospital also participated in teaching the students. This involvement was seen to be empowering. The interviewees felt it fostered the development of a teaching culture and the integration of teaching and service in the hospital.

FP02-2012 I think we were very fortunate in that it was never my sole responsibility to look after the students. The rest of the team, or most of them, have a teaching spirit at heart. It’s just one of those things that was one of the unforeseen benefits of this team is that they all have this mentality of enjoying to work with students or junior staff members, able to transfer knowledge.

### Enablers and Challenges for Training on the District Health Platform

The opportunity to teach was enabling for the family physicians. The unstructured nature of the work on the platform created unique learning opportunities, but was also seen as challenging, because the family physicians experienced uncertainty about their approach. They also described the lack of time to teach as a frustration.

**Opportunity to teach:** For the family physicians, the opportunity to participate in the academic program led to the updating of their own clinical knowledge. They further valued the opportunities for capacity development, the outreach support and the nurturing relationships with the students.

FP03-2012 A strong point for us as doctors, a good point is that we now get up to date again with stuff. So we can also ask them (the students) and we learn from them. (T)

FP02-2012 Yes, and I think the other thing that was also an enabler was the outreach of the consultants from Y (regional hospital) to Z (district hospital). (T)

FP04-2012 There is a sense of community in small areas like Z. So we tend to value people and we tend to want to know what each person has to offer, and we tend to want everybody to be welcome and to be part of the family.

**Structure and organization:** The interviewees thought that the unstructured nature of work on the district platform and the openness of the ‘curriculum walking through the door’ created opportunities for learning, but also posed challenges. They realized that they needed to adapt in order to deal with the daily disruptions.

FP02-2014 How to create a structure in an environment that actually doesn’t have structure, because everything that happens there is very dynamic. (T)

FP05-2014 One thing that makes the longitudinal model a challenge is that the objectives are relatively vague, if we’re talking now about learning outcomes. It’s very broad and we haven’t thought at all yet in terms of a curriculum of what must be covered. So it gives me tremendous freedom, which is nice, but that lack of structure also makes it sort of that sometimes you’re going to have a lot of productive outreaches and at other times relatively little is going to happen. (T)

**Uncertainty:** Dealing with uncertainty emerged as a key challenge. The family physicians were uncertain whether they were teaching the “right things”, and whether the students’ learning outcomes were being met. They worried that they were perhaps not up to date with theoretical knowledge, but felt confident about their practical experience and skills. This concern was exacerbated by the students’ anxiety about their examinations.

FP03-2012 I’m always scared, because my things are already out of date and you find your own way of doing something, don’t you? Then I’m always afraid if I’m going to say in my way how they have to do something, or explain and so on, it’s maybe inconsistent with their notes, or like X (tertiary hospital). (T)

FP05-2014 The students enjoy the program tremendously, due to the practical exposure and the responsibility that they get, and then there’s the fear that they aren’t getting the same amount of academic input as the X (tertiary hospital) and the Y (secondary hospital) students, and how are they going to do in the exams. (T)

Despite the family physicians’ uncertainties and concerns about the LIC model, it ultimately exceeded their expectations and was easier to manage than anticipated. Over time, they became more familiar with the model, adapted to the challenges, and enjoyed their teaching role.

FP05-2014 So what was initially a big challenge for me, and [one that] I welcomed with uncertainty, I later really liked it. I can remember especially certain days, that I really go out with uncertainty and then I come back and then I say yes, it’s wonderful, I have the best job in the world. (T)

**Time:** The availability of dedicated time for teaching in a busy district hospital is a scarce commodity and is threatened by competing demands emerging from clinical workloads, patients, students and management responsibilities. The interviewees felt that there should be dedicated and protected time for teaching.

FP02-2014 It’s unfortunately the nature of a district hospital, and also my work, [that] there are always other flashpoints too or meetings or other things where I’m also involved, and that we can’t necessarily always bargain on that time. (T)

FP06-2015 Yes, it’s a time thing. Despite, so even this year that I really try to make it … more time for it, it’s still maybe even difficult. Still difficult not to … for them … They also have such [a] full program, yes, to focus on the things that they have to do now. (T)

## Discussion

It is evident that the family physicians saw the rural district health platform as an ideal place to train medical students. Students learnt from an appropriate patient profile based on common conditions and first-contact care. They acquired an approach to undifferentiated care, became competent in procedural skills, and took part in community-based care. The longitudinal nature of the training added the additional benefits of patient-centered learning and continuity of care, while students acquainted themselves with the health facility staff, and the community and its health needs. As shown in earlier studies, this fostered the development of positive relationships and acceptance of the students as valued members of the healthcare team [20].

The family physicians found the teaching role both rewarding and stimulating. This was the case despite uncertainties regarding fulfilling this role effectively, anxieties with regard to student success, and challenges relating to maintaining a workable balance between their different responsibilities. Similar results were reported in reviews of the international literature [20,21]. The family physicians’ personal and professional development as a result of supervising the students also had positive implications for service delivery, an outcome supported by other studies [12,23].

Notwithstanding this positive picture, the findings of this study bring to light constraints and challenges faced by this model of training. There are important issues that need to be taken into account when seeking to extend the training of students into rural districts. Having to deal with uncertainty was an issue for the family physicians, since they initially had not felt equipped for the task and they were unsure whether the students’ learning outcomes were being met. This is not an unexpected phenomenon, as training in new settings using different learning strategies is often a source of uncertainty [12,23,24]. The family physicians were particularly concerned about the students’ anxiety about doing the same discipline-based assessment as their peers training at the tertiary hospital [20]. Although there is ample evidence that students training on a rural platform perform as well as – and sometimes better – than their peers at the academic hospital, this message of positive academic outcomes does not appear to have reached the students themselves [22,26].

Work in rural district health services is fast-paced, and without an established tradition of training. For the learner moving from a highly structured tertiary learning environment, this requires a mind shift [24]. Dube et al describe students “experiencing moments of absolute confusion” in the first months of a longitudinal clerkship, which necessitated action to restore balance [25]. Competing training and workload demands complicate the situation, although this is not unique to rural district health services.

The family physicians were of the opinion that the students made a contribution to the health services as well as to the community. More concrete evidence of this response needs to be gathered to convince health service managers of the advantages of providing dedicated time for training at their health facilities and to address anxieties about physician productivity [23].

**Figure 1:**
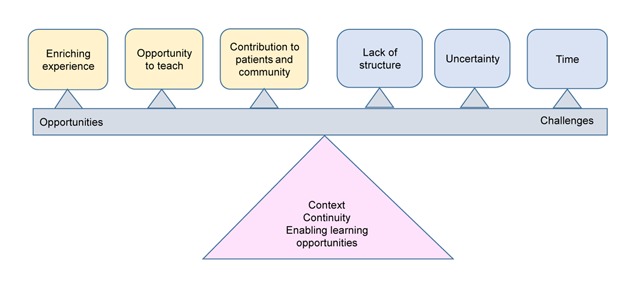
Balancing Opportunities and Constraints for Teaching at the Rural District Health Complex.

The flexibility of the integrated approach to patient care on the rural district complex platform in itself facilitates an abundance of learning opportunities and allows the students to direct their own learning [20]. However, nurturing relationships are needed for students to develop confidence to work in this complex environment. The family physicians themselves need relevant orientation, development, and support from the central campus to assist them in establishing a culture of training at the rural district complex. Von Pressentin et al. developed a concept of structured learning for this platform based on the identification of signposts for learning, a framework for weekly training activities, and focused teaching [24]. Establishing such a framework for learning on the rural district health platform is essential for meeting the students’ anticipated learning outcomes [24]. Figure 1 shows the dynamic balance between the opportunities and challenges for teaching and learning on the rural district health platform. The interplay between these factors is not static and is best managed through understanding their context and by enhancing the prospects offered by the training platform.

In this study, the context of the rural district platform, and specifically the rural hospital, framed the experiences of the family physicians, the students, and their learning. This is demonstrated in Figure 1 where the centrality of the context actually forms the pivot around which the constraints and the opportunities rotate. The figure, for example, illustrates the rich learning opportunities offered by the context, but, on the other hand, it identifies the constraints, such as the lack of time and unstructured nature of the work. The findings of this study therefore support a place-based pedagogy, given that “the context of learning is one of the most important factors that determine the outcome of learning” [27]. Reid has argued that the formation of close relationships leading to meaningful teamwork is typical of smaller, more close knit rural/decentralized communities [27]. The family physicians were therefore able to welcome the students into the team and adopt them as their own – a process which in turn influenced the students’ confidence and performance.

Although the interviewees in this study represent the full cohort of family physician supervisors in this particular program, it is acknowledged that the results reflect the experiences of only a small group in the Western Cape. Nevertheless, it has been argued that studies – which use multiple interviews with the same participants — require fewer participants [28]. Either way, more research in this area is needed. For example, the perspectives of other stakeholders, such as health facility staff, managers, and the community, would further enrich – and potentially validate – the findings.

## Conclusion

The role of the family physician in Africa in the context of primary care settings, including their capacity building and teaching role, has received considerable attention in the literature in recent years [13,14,15,16,17]. The African family physician is therefore well positioned to make an important contribution to the upscaling of decentralized training of medical and other health professions students. However, it will be important for academic institutions and district health district services to manage the balance between opportunities and challenges that present themselves in the rural context, and to nurture, support, and enable these young pioneers.
